# Peptide Toxins in Solitary Wasp Venoms

**DOI:** 10.3390/toxins8040114

**Published:** 2016-04-18

**Authors:** Katsuhiro Konno, Kohei Kazuma, Ken-ichi Nihei

**Affiliations:** 1 Institute of Natural Medicine, University of Toyama, Toyama 930-0194, Toyama, Japan; cokazuma@inm.u-toyama.ac.jp; 2 Faculty of Agriculture, Utsunomiya University, Utsunomiya 321-8505, Tochigi, Japan; nihei98@cc.utsunomiya-u.ac.jp

**Keywords:** solitary wasp, peptide toxin, neurotoxin, cytolytic peptide, bradykinin-related peptide, FMRFamide neuropeptide

## Abstract

Solitary wasps paralyze insects or spiders with stinging venom and feed the paralyzed preys to their larva. Accordingly, the venoms should contain a variety of constituents acting on nervous systems. However, only a few solitary wasp venoms have been chemically studied despite thousands of species inhabiting the planet. We have surveyed bioactive substances in solitary wasp venoms found in Japan and discovered a variety of novel bioactive peptides. Pompilidotoxins (PMTXs), in the venoms of the pompilid wasps *Anoplius samariensis* and *Batozonellus maculifrons*, are small peptides consisting of 13 amino acids without a disulfide bond. PMTXs slowed Na^+^ channel inactivation, in particular against neuronal type Na^+^ channels, and were rather selective to the Na_v_1.6 channel. Mastoparan-like cytolytic and antimicrobial peptides are the major components of eumenine wasp venoms. They are rich in hydrophobic and basic amino acids, adopting a α-helical secondary structure, and showing mast cell degranulating, antimicrobial and hemolytic activities. The venom of the spider wasp *Cyphononyx fulvognathus* contained four bradykinin-related peptides. They are hyperalgesic and, dependent on the structure, differently associated with B_1_ or B_2_ receptors. Further survey led to the isolation of leucomyosuppressin-like FMRFamide peptides from the venoms of the digger wasps *Sphex argentatus* and *Isodontia harmandi*. These results of peptide toxins in solitary wasp venoms from our studies are summarized.

## 1. Introduction

The Hymenoptera is one of the largest orders among insects. It is composed of at least 200,000 species of bees, wasps and ants. Most of them have stinging venom, utilized for prey capture and self-defense. There are two distinct lifestyles among the Hymenopteran insects: social and solitary life. Social Hymenoptera includes honeybee, hornets, paper wasps and ants, whereas solitary Hymenoptera includes solitary wasps and parasitic wasps.

Social bees and wasps use their venom for defending themselves and their colonies from attacks by enemies and predators. Stinging with these venoms produces local pain and damage, and occasionally death, in large vertebrates including man. The chemical constituents of these venoms were well documented many years ago: biogenic amines, peptides and proteins act together to produce the toxic and biological effects [1,2]. In contrast, solitary wasps offensively use their venoms for prey capture. They inject their venoms to insects or spiders and paralyze the prey to feed their larvae. Therefore, the solitary wasp venoms should contain neurotoxins acting on nervous systems, ion channels and neuronal receptors in some ways [3]. Until recently, however, only a few studies have been reported for chemical components in solitary wasp venoms. It may be because of the extreme difficulty in collecting the number of specimens required to get a sufficient amount of venom to do bioassay-guided fractionation and chemical analysis due to their solitary lifestyle.

Pioneering studies demonstrated that neurotoxins are indeed contained in solitary wasp venoms. The first neurotoxic component characterized in solitary wasp venom was kinins. In 1987, Piek *et al.* isolated threonine^6^-bradykinin (Thr^6^-BK) and megascoliakinin (Thr^6^-BK-Lys-Ala) from the venoms of the European scoliid wasps *Megascolia flavifrons* and *Colpa interrupta* [4,5]. These kinins irreversibly block the synaptic transmission of the nicotinic acetylcholine receptor (nAChR) in the insect central nervous system [5,6]. Shortly thereafter, philanthotoxins (PhTXs) were found in the venom of the African digger wasp *Philanthus triangulum* [7,8]. They are acylpolyamine toxins and non-competitive antagonists of the glutamate receptor (GluR) and nAChR [9,10].

We collected solitary wasps found in Japan, and surveyed bioactive compounds in their venom, focusing on small molecules and peptides. We first found the novel peptide neurotoxins pompilidotoxins (PMTXs) in pompilid wasp venoms [11,12], and further survey on other venoms led to the isolation of antimicrobial and cytolytic peptides, a novel type of bradykinin-related peptides and FMRFamide peptides. Thus, our studies indicated that solitary wasp venom might contain a variety of bioactive peptides besides neurotoxins, which could be potentially useful for drug discovery. In this mini-review, we summarize the results from our studies on peptide toxins in solitary wasp venoms.

## 2. Pompilidotoxins

**Figure 1:**
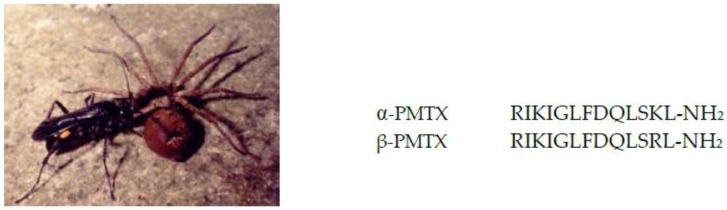
The spider wasp *Anoplius samariensis*, taking the paralyzed prey to her nest.

The spider wasp *Anoplius samariensis*, hunting only spiders as shown in Figure 1, is one of the most abundant species among the solitary wasps inhabiting in Japan. We isolated a peptide neurotoxin, α-PMTX, from the venom of this wasp by bioassay-guided fractionation using a lobster leg neuromuscular junction. The venom of another spider wasp, *Batozonellus maculifrons*, also contained α-PMTX together with the closely related β-PMTX, in which the Lys residue at position 12 was replaced by Arg [11]. Both toxins greatly facilitated neurotransmission in the lobster leg neuromuscular junction [13], and β-PMTX is five times more potent than α-PMTX. Structure-activity relationships studies using a number of synthetic analogs of α-PMTX with the lobster neuromuscular synapse showed that the basic amino acids at positions 1, 3 and 12 were important, and the potency was enhanced three to five times when they were replaced by the other, Lys–Arg or Arg–Lys. Furthermore, the *C*-terminal amide structure and the length of the 13 amino acids are essential since the *C*-terminal carboxyl analog and any analogs with shorter amino acid lengths were reduced or lost activity [14].

PMTXs act not only on crustacean neuromuscular synapses but also on mammalian nervous systems such as rat cortical neurons and hippocampal neurons [15,16,17]. Electrophysiological experiments revealed in detail that PMTX blocks voltage-gated sodium channels (VGSC) by slowing inactivation [18,19], and it discriminates between neuronal and cardiac VGSC, acting only on neuronal Na channels [20]. Accordingly, we conducted a thorough study of the effects of PMTX on VGSC isoforms, including seven mammalian VGSC isoforms (Na_v_1.1—Na_v_1.7) and one insect VGSC (DmNa_v_1), by evaluating the sodium current inactivation [21]. No effects were seen for Na_v_1.4 and Na_v_1.5, moderate effects were seen for Na_v_1.1—Na_v_1.3 and Na_v_1.7, and the most potent effect was seen for Na_v_1.6; in other words, it is rather selective to Na_v_1.6. The effects for the insect VGSC DmNa_v_1 were far more potent than that for Na_v_1.6, which is reasonable since the stinging target of solitary wasps is mostly insects.

Toxins targeting sodium channels similar to PMTXs are widely distributed in animal venoms [22], in particular contained as major toxins in sea anemone, scorpion and spider venoms [22,23,24,25,26]. These toxins play an important role in prey capture and self-defense. On the other hand, they have been used as research tools for neuronal functions, such as elucidating action mechanisms of ion channels or characterizing receptor functions, and some of them are of clinical interest for neurological disorders. PMTXs are small peptides consisting of only 13 amino acids without a disulfide bond, which is in marked contrast to other toxins with 40–60 amino acids and three to four disulfide bonds. Therefore, PMTXs may also be useful for research tools and drug discovery [27,28,29,30,31,32].

## 3. Antimicrobial and Cytolytic Peptides

The Eumenine wasp *Anterhynchium flavomarginatum micado* may be the most common solitary wasp in Japan, hunting only green caterpillars and building their nests in bamboo twigs with mud. The major component of this venom, eumenine mastoparan-AF (EMP-AF), is a peptide structurally related to mastoparan [33]. Mastoparan was initially isolated from hornet venom, then closely related peptides were found in a variety of social wasp venoms (hornets and paper wasps) [2]. Accordingly, they are collectively called mastoparans or mastoparan-like peptides. The mastoparans are 14 amino acids in length with an amidated *C*-terminus, and they are rich in hydrophobic and basic amino acids, which makes these peptides have an amphipathic chemical character, adopting an α-helical secondary structure under proper conditions. This chemical feature is essential for their biological activities through the cell membrane, and accordingly, they are also called cytolytic peptides. EMP-AF has all the chemical characteristics of mastoparans, and, in fact, showed high sequence similarity to mastoparan [33] and a high content of α-helical conformation under SDS (sodium dodecyl sulfate) micelle and TFE (trifluoroethanol) conditions by circular dichroism (CD) spectral analysis [34]. NMR analysis supported the CD results, and also showed that the *C*-terminal amide stabilized the α-helical conformation [35]. In biological evaluation, EMP-AF stimulated degranulation from rat peritoneal mast cells and RBL-2H3 cells to an extent similar to mastoparan, and showed significant hemolytic activity in human erythrocytes. Antimicrobial activity was also investigated because cytolytic peptides are known to show antimicrobial activity. EMP-AF exhibited potent growth inhibition against Gram-positive bacteria as compared to Gram-negative bacteria, for example IC_50_ was 5 g/mL to *Staphylococcus aureus* ATCC6538 [34].

Eumenine solitary wasps may have such antimicrobial and cytolytic peptides in common, but their structures and biological activities are somewhat different depending on the species. Those discovered so far are summarized in Table 1. Eumenine mastoparan-OD (EMP-OD) is the second mastoparan to be found in the Eumenine wasp venom of *Orancistrocerus drewseni drewseni*. This novel peptide has typical chemical features of mastoparan, and exhibits more potent hemolytic activity than that of mastoparan [36,37]. Eumenitin, from *Eumenes rubronotatus*, is 15 amino acids in length with an extra hydrophilic amino acid at the *C*-terminus and is not amidated; it still has basically the same chemical features as those of mastoparans. It showed the same biological activities as mastoparans, but its potency tends to be lower [38,39]. Three other eumenine wasp venoms have both mastoparan-type and Eumenitin-type peptides together: EMP-ER and Eumenitin-R from *Eumenes rubrofemoratus*; EMP-EF and eumenitin-F from *Eumenes fraterculus* [40]; EpVP2a and EpVP1 from *Eumenes pomiformis* [41]. Eumenitin-type peptides have only been contained in solitary wasp venoms, and never found in social wasp venoms. Decoralin from *Oreumenes decorates* is a smaller peptide, being only 11 amino acids in length, but the chemical features can be classified into Eumenitin-type. In fact, it showed typical biological activities of cytolytic peptides [42].

In addition to Eumenine wasps, an antimicrobial and cytolytic peptide has been found in the spider wasp. Anoplin was isolated as a minor component from *Anoplius samariensis* venom, which contained α-PMTX as a major component [43]. This peptide has only 10 amino acids, but both chemical and biological features were typical for that of cytolytic peptides [44]. Interestingly, the similarities and differences of anoplin *vs.* decoralin correspond to those of mastoparan *vs.* eumenitin. Anoplin is the smallest peptide among the antimicrobial and cytolytic peptides found from natural sources, which may make it a lead compound for developing new, potent and useful antimicrobial compounds [45,46,47,48].

Antimicrobial and cytolytic peptides are widely found in many other arthropod venoms such as spider and scorpion venoms [49]. The majority of these peptides are linear, cationic and amphipathic in character, but the amino acid length is variable from 15 to 60 residues. They may play a dual role in acting as antimicrobials and potentiating the venom toxicity by disturbing excitable membranes. It can also be the case for the solitary wasp venoms [50]. Antimicrobials are injected beforehand for protection against microbial infection when larvae consumes the paralyzed prey.

## 4. Bradykinin-Related Peptides

In 1987, the first structure of a solitary wasp toxin was reported. Two bradykinin-related peptides, threonine^6^-bradykinin (Thr^6^-BK) and megascoliakinin (MBK), were isolated from the venoms of the European scoliid wasps *Megascolia flavifrons* and *Colpa interrupta* [4,5], as mentioned in the Introduction. The first toxin found in social wasp venoms was also a bradykinin-related peptide found as a pain-producing substance in 1954, and since then, many bradykinin-related peptides, collectively called wasp kinins, have been isolated and chemically characterized from Polistes and Vespid social wasp venoms [2]. Wasp kinins show similar properties to bradykinin (BK) both chemically and pharmacologically, containing a whole BK sequence elongated at the *C*- or *N*- terminal, and showing hypotensive and inflammatory activities. Piek *et al.* reported that Thr^6^-BK and megascoliakinin irreversibly block the synaptic transmission of the nicotinic acetylcholine receptor (nAChR) in the insect central nervous system, which indicates that these kinins play a major role in the paralyzing activity of the solitary wasp venoms [5,6].

**Figure 2:**
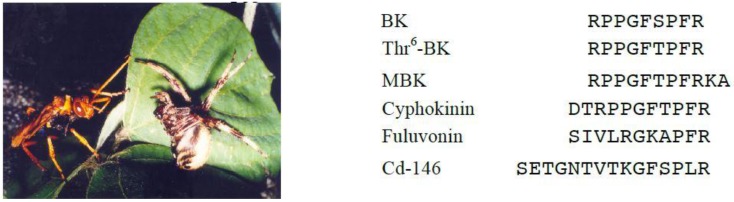
The spider paralyzed by the stinging venom of *Cyphononyx fulvognathus.*

Piek *et al.* further investigated the pharmacological activities of solitary wasp venoms and found that scoliid wasp venoms commonly showed kinin activity [51]. We demonstrated the presence of Thr^6^-BK in the venoms of Japanese scoliid wasps [52]. Screening of 23 venom extracts from Japanese solitary wasps by MALDI-TOF MS revealed that scoliid wasp venoms of *Megacampsomeris prismatica*, *Campsomeriella annulata annulata* and *Carinoscolia melanosoma fascinata* contained Thr^6^-BK as one of the major components. This peptide was also identified in the venom extracts of the spider wasp *Cyphononyx fluvognathus* (formerly *Cyphononyx dorsalis*, Figure 2) [53]. This was the first example where a bradykinin-related peptide was found in spider wasp venom. We therefore were interested in this venom and analyzed the minor components carefully, which resulted in finding novel bradykinin-related peptides [54]. Cyphokinin may be classified into wasp kinins because it includes the whole sequence of Thr^6^-BK with only two extra amino acid residues elongating at its *N*-terminus. On the other hand, Cd-146 and fulvonin show a lesser similarity to the bradykinin sequence. Cd-146 has a kinin-like sequence at the *C*-terminal with six residues, while fulvonin has only three residues identical to BK. In pharmacological assays, only cyphokinin, including the whole sequence of BK, promoted the contraction of guinea pig ileum smooth muscle preparations, which was completely blocked by HOE-140, the B_2_ receptor antagonist, whereas only fulvonin potentiated BK-elicited smooth muscle contraction. In the rat paw pressure test after intraplantar injection, all the peptides showed a hyperalgesic effect with different strengths. This effect is due to the action of BK receptors, since the hyperalgesia induced by Cd-146 and fulvonin was blocked by the B_1_ receptor antagonist, while the effect of cyphokinin was reversed by the B_2_ antagonist. Thus, these BK-related peptides are a new type of wasp kinins and show distinct biological activities associated with BK receptors.

The component responsible for the paralytic activity of the *C. dorsalis* venom was reported to be a protein. Bioassay-guided fractionation led to the identification of an arginine-kinase-like protein with high homology to that of honeybee. Recombinant protein expressed in *E. coli* exhibited paralytic activity against spiders with the same characteristic symptoms as the crude venom [55]. Arginine-kinase-like protein was also found in the two Eumenine wasp venoms of *Eumenes pomiformis* and *Orancistrocerus drewseni* as the most predominant protein in both venoms [56]. The arginine-kinase of these solitary wasps had high sequence identities to those of *C. dorsalis* (95%–96%), and the honeybee *A. mellifera* (86%–88%).

## 5. FMRFamide-Related Neuropeptides

Digger wasps are a large group of solitary wasps, and they build a nest by digging a burrow in the ground. They have a large variety of hunting prey from spiders and crickets to flies and bees. *Philanthus triangulum*, inhabiting the Sahara desert in Africa, is the only species among digger wasps that have been chemically studied, and their major toxin is philanthotoxins-433 (PhTX-433) [7,8], as mentioned in the Introduction. This wasp toxin has been utilized for extensive studies on glutamatergic neurotransmission systems, resulting in the discovery of highly useful glutamate receptor antagonists [57].

**Figure 3:**
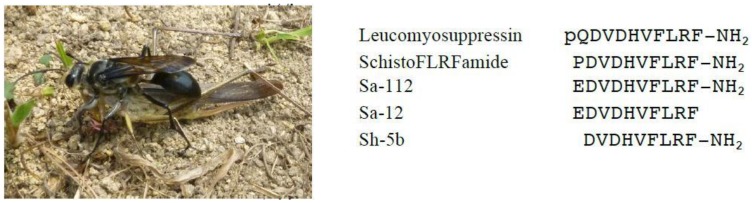
The digger wasp *Sphex argentatus argentatus* is stinging the grasshopper prey.

Many digger wasps inhabit Japan, but they have never been studied until quite recently. We have chemically studied the venoms of two dominant species in Japan, *Sphex argentatus argentatus* and *Isodontia harmandi*, and found FMRFamide-related neuropeptides for the first time in solitary wasp venom [58]. *Sphex argentatus argentatus* is frequently found and is the largest in size among the Japanese solitary wasps. They sometimes make a colony, nesting in the same place, and hunt only grasshoppers as shown in Figure 3. From the venom extracts of this wasp, we isolated two closely related peptides, Sa-112 and Sa-12,both of which have the same 10-amino-acid sequence and the only difference is whether the *C*-terminal is amidated or not. Sa-112 was also found in the venom of the close relative *Isodontia harmandi*, from which Sh-5b, an *N*-terminal-missing analog of Sa-112, was obtained together. All these peptides should be classified into FMRFamide-related peptides because they bear the FMLF sequence at the *C*-terminal. The FMRFamide peptides are called due to their characteristic *C*-terminal sequence, and they are a group of neuropeptides, distributed widely in arthropods and in insects, involved in the regulation of physiological processes by acting as neurotransmitters, neurohormones and/or neuromodulation. These processes generally include activities such as digestion, circulation, and reproduction [59]. Sa-112 is highly homologous to leucomyosupressin [60] and SchistoFLRFamide [61], the FMRFamide-related neuropeptides from the cockroach and locust, respectively. Only the *N*-terminal amino acid was replaced to E (glutamic acid) from P (proline) or pQ (pyroglutamic acid), and accordingly, it is expected to show similar activities to those of the known neuropeptides. Indeed, Sa-112 inhibited contraction of the locust oviduct, a typical activity of FMRFamide peptides, at 50 nM to the same extent as SchistoFLRFamide, while Sa-12, a non-amidated peptide, showed no activity in the locust oviduct contraction. These results demonstrated again the essential role of the *C*-terminal amide structure to their biological activity. This is the first example of an FMRF-related neuropeptide to be found in solitary wasp venom. Another peptide, Sh-5b, was already synthesized for structure-activity relationship studies and was shown to have the same activity as that of SchistoFLRFamide. 

It is interesting that these digger wasps inject FMRFamide neuropeptides into their prey, grasshoppers, because the grasshoppers have similar neuropeptides in their body and they are functioning in some way. Then, what is the role and effect of injected neuropeptides from wasp venom? They may not be directly involved in the paralyzing effect, but might play a supporting role in disturbing physiological regulation with the neuropeptide in the grasshoppers. In any case, it should be studied further.

## 6. Other Peptide Toxins

We have found other novel peptides in spider wasp venom. They are Cd-125 from *Cyphononyx dorsalis* [53], As-126 from *Anoplius samariensis* [62] and Bm-10 from *Batozonellus maculifrons* [62] as summarized in Table 2. The sequences were determined mainly by using mass spectrometric methods. In particular, Cd-125 is the major component of this wasp venom with a length of only 8 amino acids, but its biological activity remains to be studied. This is also the case for As-126 and Bm-10, the minor components of these venoms.

Orancis-Protonectin was isolated from the Eumenine wasp venom of *Orancistrocerus drewseni drewseni* together with EMP-OD [36,37]. The sequence is highly homologous to those of protonectins, hemolytic peptides isolated from social wasp venoms. In fact, Oricis-Protonectin showed more potent hemolytic activity than mastoparan. This is the first example of a protonectin-type peptide that was found in solitary wasp venom.

The presence of a higher molecular weight peptide, As-fr-19, in the *Anoplius samariensis* venom has been reported [63]. It was found in a fraction responsible for spider paralysis. This peptide is a novel multiple-cysteine peptide with high sequence similarity to known peptides such as dendrotoxins (K^+^ channel blocker) and Kuniz-type protease inhibitor. However, its biological properties are not characterized yet because it is still a component of a fraction (a mixture of several peptides). A dendrotoxin-like sequence was also found in the transcripts from the Eumenine wasp venoms [41].

## 7. Concluding Remarks

The major role of solitary wasp venom is to paralyze the prey and serve it to the wasps’ larva as food. Therefore, the solitary wasp venoms should contain neurotoxins acting on a variety of nervous systems. Our studies indicated that solitary wasp venoms might contain a variety of bioactive peptides besides neurotoxins, such as antimicrobial and cytolytic peptides, bradykinin-related peptides and neuropeptides. As discussed by Lee and co-workers [64], there are similarities and differences between solitary and social wasp toxins. Antimicrobial and cytolytic peptides and bradykinin-related peptides are common, but pompilidotoxins and FMRFamide-related neuropeptides are distinct to solitary wasp venoms. The prey of solitary wasps is specific to the class of wasps, for example pompilido wasps prey only on spiders, whereas eumenine wasps only on caterpillars. The reasons for this specificity are not known yet, but it may indicate that distinct components are included in the solitary wasp venoms depending on the species and/or class of wasps. Therefore, more diverse bioactive substances would be found if more solitary wasp venoms were studied. On the other hand, those solitary wasp toxins would be potentially useful not only for research tools but also for drug discovery, along with other venom toxins from snakes, scorpions, spiders and cone snails. It is noteworthy that the structure of these peptide toxins is relatively simple: only 8–16 amino acids in length without a disulfide bond, which is advantageous for practical use and development. Among more than 20,000 solitary wasps inhabiting in the planet, only about 10 species have been studied thus far. It may be because of the extreme difficulty in collecting a sufficient amount of the venom; in other words, a large number of wasps is required for bioassay-guided fractionation and chemical analysis due to their solitary lifestyle. However, recent developments of chemical analysis, especially by mass spectrometry, have made it possible to analyze quite minute amounts of venom components [53,62,65]. Quite recently, we established that, with using LC-MS, only 10% of the amount of crude venom from a single wasp specimen is sufficient to analyze most of the peptide toxins (peptidomic analysis). LC-MS/MS analysis allows *de novo* sequencing of most of the peptides, sometimes with more than 100 components, contained in the crude venom [66]. Biological activities would be evaluated by using synthetic specimens of deduced sequences. This is in marked contrast to bioassay-guided fractionation, which gives only major and a few minor components. With this method in hand, we are further surveying solitary wasp venoms, which would lead to finding a variety of useful bioactive substances.

## Figures and Tables

**Table 1:** Antimicrobial and cytolytic peptides in solitary wasp venoms.

Name	Sequence	Name	Sequence
Mastoparan	INLKALAALAKKIL-NH_2_	EMP-OD	GRILSFIKGLAEHL-NH_2_
EMP-AF	INLLKIAKGIIKSL-NH_2_	Eumenitin	LNLKGIFKKVKSLLT
EMP-ER	FDIMGLIKKVAGAL-NH_2_	Eumenitin-R	LNLKGLIKKVASLLN
EMP-EF	FDVMGIIKKIASAL-NH_2_	Eumenitin-F	LNLKGLFKKVASLLT
EpVP2a	FDLLGLVKKVASAL-NH_2_	EpVP1	INLKGLIKKVASLLT
Anoplin	GLLKRIKTLL-NH_2_	Decoralin	SLLSLIRKLIT

**Table 2:** Miscellaneous peptide toxins in solitary wasp venoms.

Name	Sequence
Cd-125	DTARLKWH
As-126	pQDPPVVKMK-NH_2_
Bm-10	pQTAPVPKAISK-NH_2_
Orancis-Protonectin	ILGIITSLLKSL-NH_2_
